# Guanidylation and Tail Effects in Cationic Antimicrobial Lipopeptoids

**DOI:** 10.1371/journal.pone.0041141

**Published:** 2012-07-23

**Authors:** Brandon Findlay, Paul Szelemej, George G. Zhanel, Frank Schweizer

**Affiliations:** 1 Department of Chemistry, University of Manitoba, Winnipeg, Manitoba, Canada; 2 Department of Medical Microbiology, University of Manitoba, Winnipeg, Manitoba, Canada; University of Helsinki, Finland

## Abstract

**Background:**

Cationic antimicrobial peptides (CAMPs) are attractive scaffolds for the next generation of antimicrobial compounds, due to their broad spectrum of activity against multi-drug resistant bacteria and the reduced fitness of CAMP-insensitive mutants. Unfortunately, they are limited by poor *in vivo* performance, including ready cleavage by endogenous serum proteases.

**Methodology/Principal Findings:**

To explore the potential for peptoid residues to replace well studied CAMP scaffolds we have produced a series of antimicrobial lipopeptoids, with sequences similar to previously reported lipopeptides. The activity of the peptoids was assessed against a panel of clinically relevant and laboratory reference bacteria, and the potential for non-specific binding was determined through hemolytic testing and repeating the antimicrobial testing in the presence of added bovine serum albumin (BSA). The most active peptoids displayed good to moderate activity against most of the Gram positive strains tested and moderate to limited activity against the Gram negatives. Antimicrobial activity was positively correlated with toxicity towards eukaryotic cells, but was almost completely eliminated by adding BSA.

**Conclusion/Significance:**

The lipopeptoids had similar activities to the previously reported lipopeptides, confirming their potential to act as replacement, proteolytically stable scaffolds for CAMPs.

## Introduction

Bacteria resistant to our current front-line therapeutics have been found sealed in permafrost, predating both the current antibiotic age and human society in general, proving antibiotic resistance mechanisms are ancient [1], [2]. Dispersed throughout the pangenome at low population levels, the widespread use of antibiotics in recent times has selected for these bacteria and their resistance mechanisms, allowing them to displace their more susceptible brethren or confer their advantage to more pathogenic strains via lateral gene transfer [3].

Widespread use of antimicrobials will therefore inevitably lead to correspondingly pervasive bacterial resistance, as genes coding for resistance to the next generation of antibiotics are already present throughout nature [1]. However, the low prevalence of resistance mechanisms in pathogenic bacteria prior to the development of commercial antibiotics may offer a means of remaining one step ahead of infectious disease. In the absence of antibiotic selective pressure, resistance mechanisms are unlikely to enhance fitness, as each superfluous drug inactivating enzyme or efflux pump requires resources which could have been used for growth and replication of the host cell [4]. In antibiotic free, nutrient poor environments resistance naive bacteria can use this edge to outcompete strains expressing resistance elements and form the dominant bacterial population. Developing antimicrobials with energy intensive or mal-adaptive resistant mechanisms may allow researchers to accentuate this fitness penalty, preventing significant levels of resistance from persisting in the absence of antibiotic use.

Cationic antimicrobial peptides (CAMPs) have demonstrated this fitness gap [5], and their persistent activity in otherwise drug resistant strains has drawn interest [6]–[8]. Counter to the “magic bullet” ideal of classical antibiotics, CAMPs interfere with a large number of targets, including negatively charged DNA and RNA, hydrophobic chaperone proteins, and the negatively charged bacterial membrane [8]. Because cell death does not result from a single interaction or pathway and is derived from the physiochemical properties of the CAMP instead of specific structural features, it can be difficult for bacteria to develop widespread CAMP resistance. While several cases of *in vitro* resistance development have been reported [5], [7], [9], resistance may lead to reduced fitness in the absence of CAMPs, due to large scale alteration of the lipid bilayer composition [5]. This is consistent with studies on resistance mechanisms in nature, which often reduce bacterial pathogenicity [9].

The key to CAMP activity is the spatial separation of opposing hydrophobic and cationic domains, which allows the CAMPs to effectively insert themselves into the negatively charged bacterial membrane, among other targets [6]. This structural plasticity allows semi-synthetic analogues as small as three residues in length to exert antimicrobial activity, but also leads to high levels of toxicity and tight binding to hydrophobic proteins such as serum albumin [10], [11]. Modifying the hydrophobic domain of the CAMPs appears to have the greatest effect on these nonspecific interactions, and linking lipid tails to short peptide sequences allows convenient analogue synthesis and rapid elucidation of optimal physiochemical properties [12].

However, the inherent protease susceptibility of CAMPs may limit their use as therapeutics, as it offers a convenient handle for resistance development [11]. Modified amino acid residues like peptoids are cleavage resistant, and have been recently used in the construction of a number of CAMP derivatives [13], [14]. Peptoid residues are structurally similar to amino acids, but have the R-group transferred from the α-carbon to the amide nitrogen. Lacking the ability to form backbone hydrogen bonds, peptoids do not form standard peptide secondary structures but able to mimic CAMP activity when composed of amphiphilic residues [13]. Having constructed a series of ultrashort antimicrobial lipopeptides [15], we set out to prepare a series of ultrashort amphiphilic peptoids to better understand the effect of the modified backbone.

## Results

Nineteen lipopeptoids were prepared according to previously published procedures (**Supporting Information S1)**, with a mixture of different sequences and lipid tails (**Table 1**). Initial synthesis of the lysine analogue containing peptoids was conducted on solid phase, with derivatization to the homoarginine containing sequences completed in solution.

**Table 1:** Lipopeptoids under consideration.

Compound Designation	Sequence	Molecular Mass
**CTAC**	N(CH_3_)_3_(CH_2_)_16_Cl	320.00 g/mol
**C11-N_lys_GN_lys_**	CH_3_(CH_2_)_9_CO-N((CH_2_)_4_NH_2_)CH_2_CO-NCH_2_CO-N((CH_2_)_4_NH_2_)CH_2_CO-NH_2_	726.75 g/mol
**C14-N_lys_GN_lys_**	CH_3_(CH_2_)_12_CO-N((CH_2_)_4_NH_2_)CH_2_CO-NCH_2_CO-N((CH_2_)_4_NH_2_)CH_2_CO-NH_2_	768.83 g/mol
**C16-N_lys_GN_lys_**	CH_3_(CH_2_)_14_CO-N((CH_2_)_4_NH_2_)CH_2_CO-NCH_2_CO-N([CH_2_]_4_NH_2_)CH_2_CO-NH_2_	796.88/mol
**C20-N_lys_GN_lys_**	CH_3_(CH_2_)_18_CO-N((CH_2_)_4_NH_2_)CH_2_CO-NCH_2_CO-N([CH_2_]_4_NH_2_)CH_2_CO-NH_2_	852.99 g/mol
**F11-N_lys_GN_lys_**	CF_3_(CF_2_)_7_CH_2_CH_2_CO-N((CH_2_)_4_NH_2_)CH_2_CO-NCH_2_CO-N((CH_2_)_4_NH_2_)CH_2_CO-NH_2_	1032.59 g/mol
**C11-N_harg_GN_harg_**	CH_3_(CH_2_)_9_CO-N((CH_2_)_4_NHCN_2_H_3_)CH_2_CO-NCH_2_CO-N((CH_2_)_4_NHC N_2_H_3_)CH_2_CO-NH_2_	810.83 g/mol
**C14-N_harg_GN_harg_**	CH_3_(CH_2_)_12_CO-N((CH_2_)_4_NHCN_2_H_3_)CH_2_CO-NCH_2_CO-N((CH_2_)_4_NHCN_2_H_3_)CH_2_CO-NH_2_	852.99 g/mol
**C16-N_harg_GN_harg_**	CH_3_(CH_2_)_14_CO-N((CH_2_)_4_NHCN_2_H_3_)CH_2_CO-NCH_2_CO-N((CH_2_)_4_NHCN_2_H_3_)CH_2_CO-NH_2_	880.96 g/mol
**C20-N_harg_GN_harg_**	CH_3_(CH_2_)_18_CO-N((CH_2_)_4_NHCN_2_H_3_)CH_2_CO-NCH_2_CO-N((CH_2_)_4_NHCN_2_H_3_)CH_2_CO-NH_2_	937.07 g/mol
**F11-N_harg_GN_harg_**	CF_3_(CF_2_)_7_CH_2_CH_2_CO-N((CH_2_)_4_NHCN_2_H_3_)CH_2_CO-NCH_2_CO-N((CH_2_)_4_NHCN_2_H_3_)CH_2_CO-NH_2_	1116.67 g/mol
**C11-N_lys_N_lys_N_lys_**	CH_3_(CH_2_)_9_CO-N((CH_2_)_4_NH_2_)CH_2_CO-N((CH_2_)_4_NH_2_)CH_2_CO-N((CH_2_)_4_NH_2_)CH_2_CO-NH_2_	1038.51 g/mol
**C14-N_lys_N_lys_N_lys_**	CH_3_(CH_2_)_12_CO-N((CH_2_)_4_NH_2_)CH_2_CO-N((CH_2_)_4_NH_2_)CH_2_CO-N((CH_2_)_4_NH_2_)CH_2_CO-NH_2_	1080.09 g/mol
**C16-N_lys_N_lys_N_lys_**	CH_3_(CH_2_)_14_CO-N((CH_2_)_4_NH_2_)CH_2_CO-N((CH_2_)_4_NH_2_)CH_2_CO-N((CH_2_)_4_NH_2_)CH_2_CO-NH_2_	1108.15 g/mol
**C20-N_lys_N_lys_N_lys_**	CH_3_(CH_2_)_18_CO-N((CH_2_)_4_NH_2_)CH_2_CO-N((CH_2_)_4_NH_2_)CH_2_CO-N((CH_2_)_4_NH_2_)CH_2_CO-NH_2_	1164.25 g/mol
**F11-N_lys_N_lys_N_lys_**	CF_3_(CF_2_)_7_CH_2_CH_2_CO - N((CH_2_)_4_NH_2_)CH_2_CO-N((CH_2_)_4_NH_2_)CH_2_CO-N((CH_2_)_4_NH_2_)CH_2_CO-NH_2_	1343.85/mol
**C11-N_harg_N_harg_N_harg_**	CH_3_(CH_2_)_9_CO-N((CH_2_)_4_NHCN_2_H_3_)CH_2_CO-N((CH_2_)_4_NHCN_2_H_3_)CH_2_CO-N((CH_2_)_4_NHCN_2_H_3_)CH_2_CO-NH_2_	911.89 g/mol
**C14-N_harg_N_harg_N_harg_**	CH_3_(CH_2_)_12_CO-N((CH_2_)_4_NHCN_2_H_3_)CH_2_CO-N((CH_2_)_4_NHCN_2_H_3_)CH_2_CO-N((CH_2_)_4_NHCN_2_H_3_)CH_2_CO-NH_2_	953.97 g/mol
**C16-N_harg_N_harg_N_harg_**	CH_3_(CH_2_)_14_CO-N((CH_2_)_4_NHCN_2_H_3_)CH_2_CO-N((CH_2_)_4_NHCN_2_H_3_)CH_2_CO-N((CH_2_)_4_NHCN_2_H_3_)CH_2_CO-NH_2_	982.03 g/mol
**C20-N_harg_N_harg_N_harg_**	CH_3_(CH_2_)_18_CO-N((CH_2_)_4_NHCN_2_H_3_)CH_2_CO-N((CH_2_)_4_NHCN_2_H_3_)CH_2_CO-N((CH_2_)_4_NHCN_2_H_3_)CH_2_CO-NH_2_	1038.13 g/mol

### Antimicrobial Activity

The cationic disinfectant **Cetyltrimethylammonium chloride** (**CTAC**, cetrimide) was used as a positive control, and displayed strong activity against Gram positive bacteria (MIC 0.5–2 µg/mL), and moderate to limited activity against Gram negative bacteria (MIC 16–128 µg/mL). *P. aeruginosa* was the least susceptible strain, in line with previous studies [16].

Activity of the lipopeptoids could be similarly divided (**Tables 2** **and** **3**), though the overall activity was lower. Activity of most peptoids was good to limited against Gram positive bacteria (MIC 8–64 µg/mL) and moderate to weak against Gram negative strains (MIC 16–512 µg/mL), with the exception of **C11-N_lys_GN_lys_** and **C11-N_lys_N_lys_N_lys_**, which were broadly inactive. Activity against *S. pneumoniae* was significantly reduced relative to other Gram positive bacteria, while all three *E. coli* strains were quite susceptible to peptoids with hydrophobic tails sixteen carbons in length. The three peptoids with fluorinated lipid tails were at best moderately active against Gram positive bacteria, slightly less effective than their C14 analogues on a mass basis.

**Table 2:** Antimicrobial testing of N_lys_GN_lys_ based lipopeptoids.

Compound Organism	CTAC	C11-N_lys_GN_lys_	C14-N_lys_GN_lys_	C16-N_lys_GN_lys_	C20-N_lys_GN_lys_	F11-N_lys_GN_lys_	C11-N_harg_GN_harg_	C14-N_harg_GN_harg_	C16-N_harg_GN_harg_	C20-N_harg_GN_harg_	F11-N_harg_GN_harg_
***S.aureus*** b	1	512	32	8	8	32	128	16	8	16	32
**MRSA** c	1	512	32	16	16	64	128	16	8	16	64
**MSSE** d	0.5	256	16	8	8	32	128	8	8	8	64
**MRSE** e	2	256	32	16	8	32	128	16	16	8	64
***E. faecalis*** f	1	512	64	16	16	64	256	32	16	16	64
***E. faecium*** g	0.5	512	64	16	16	64	256	32	16	16	32
***S.pneumoniae*** h	2	512	256	128	128	256	512	128	128	256	256
***E.coli*** i	16	>512	128	16	256	512	512	32	16	256	64
***E.coli*** j	32	>512	128	16	256	512	512	64	16	256	128
***E.coli*** k	16	>512	128	16	128	512	512	64	16	256	128
***P.aeruginosa*** l	128	>512	256	64	256	512	>512	64	64	512	256
***P.aeruginosa*** m	64	>512	512	128	256	512	512	128	64	256	256
***S. maltophilia*** n	32	>512	512	128	128	>512	>512	256	64	256	>512
***A. baumannii*** o	32	>512	256	128	128	>512	512	512	128	128	512
***K.pneumoniae*** p	16	>512	256	64	128	>512	512	256	64	128	512
**Haemolysis** q	77.05	1.34	2.90	56.03	33.51	4.84	1.49	55.44	61.06	71.87	7.45

a MIC, reported in µg/mL.

b ATCC 29213.

c ATCC 33592.

d 81388 CANWARD 2008.

e CAN-ICU 61589.

f ATCC 29212.

g ATCC 27270.

h ATCC 49619.

i ATCC 25922.

j CAN-ICU 61714.

k CAN-ICU 63074.

l ATCC 27853.

m CAN-ICU 62308.

n CAN-ICU 62584.

o CAN-ICU 63169.

p ATCC 13883.

q Percent haemolysis at 100µg/mL of compound.

**Table 3:** Antimicrobial testing of N_lys_N_lys_N_lys_ based lipopeptoids.

Compound Organism	CTAC	C11-N_lys_N_lys_N_lys_	C14-N_lys_N_lys_N_lys_	C16-N_lys_N_lys_N_lys_	C20-N_lys_N_lys_N_lys_	F11-N_lys_N_lys_N_lys_	C11-N_harg_N_harg_N_harg_	C14-N_harg_N_harg_N_harg_	C16-N_harg_N_harg_N_harg_	C20-N_harg_N_harg_N_harg_
***S.aureus*** b	1	512	32	16	16	32	128	16	8	8
**MRSA** c	1	512	64	32	16	32	128	16	16	16
**MSSE** d	0.5	512	32	8	8	32	64	8	8	8
**MRSE** e	2	512	16	8	8	32	256	16	8	8
***E. faecalis*** f	1	512	64	32	16	64	512	32	16	16
***E. faecium*** g	0.5	256	64	32	16	64	256	16	16	8
***S.pneumoniae*** h	2	>512	128	128	64	128	512	64	64	64
***E.coli*** i	16	>512	128	32	32	64	>512	64	16	64
***E.coli*** j	32	>512	128	64	32	64	>512	64	32	64
***E.coli*** k	16	>512	128	64	32	128	>512	64	32	32
***P.aeruginosa*** l	128	>512	512	128	128	128	>512	256	64	128
***P.aeruginosa*** m	64	>512	512	128	64	256	>512	128	64	128
***S. maltophilia*** n	32	>512	>512	256	64	>512	>512	256	128	64
***A. baumannii*** o	32	>512	512	256	64	>512	>512	256	128	64
***K.pneumoniae*** p	16	>512	512	128	64	>512	>512	256	256	64
***Haemolysis***	77.05	0.67	2.19	67.69	68.29	8.13	0.71	20.34	30.80	71.50

a MIC, reported in µg/mL.

b ATCC 29213.

c ATCC 33592.

d 81388 CANWARD 2008.

e CAN-ICU 61589.

f ATCC 29212.

g ATCC 27270.

h ATCC 49619.

i ATCC 25922.

j CAN-ICU 61714.

k CAN-ICU 63074.

l ATCC 27853.

m CAN-ICU 62308.

n CAN-ICU 62584.

o CAN-ICU 63169.

p ATCC 13883.

q Percent haemolysis at 100µg/mL of compound.

### Antimicrobial Activity in the Presence of BSA

The addition of 4% BSA significantly reduced the activity of **CTAC** (**Tables 4** **and** **5**), raising activity against Gram positive bacteria roughly sixteen fold (MIC 8–128 µg/mL) while all but eliminating activity against Gram negative bacteria (MIC ≥512 µg/mL).

**Table 4:** Antimicrobial testing of N_lys_GN_lys_ based lipopeptoids in the presence of 4% bovine serum albumin.

Compound Organism	CTAC	C11-N_lys_GN_lys_	C14-N_lys_GN_lys_	C16-N_lys_GN_lys_	C20-N_lys_GN_lys_	F11-N_lys_GN_lys_	C11-N_harg_GN_harg_	C14-N_harg_GN_harg_	C16-N_harg_GN_harg_	C20-N_harg_GN_harg_	F11-N_harg_GN_harg_
***S.aureus*** b	32	>512	512	512	512	256	512	512	512	512	512
**MRSA** c	32	>512	512	512	512	256	512	512	512	512	512
**MSSE** d	16	512	512	512	256	128	256	512	512	256	512
**MRSE** e	128	512	512	512	512	256	512	512	512	256	512
***E. faecalis*** f	32	>512	>512	512	512	256	>512	512	512	512	512
***E. faecium*** g	64	>512	512	512	512	256	512	512	512	256	256
***S.pneumoniae*** h	8	512	>512	512	512	512	>512	512	512	64	512
***E.coli*** i	512	>512	>512	512	>512	512	512	512	512	512	>512
***E.coli*** j	512	>512	>512	512	>512	512	>512	>512	512	>512	512
***E.coli*** k	512	>512	>512	512	>512	>512	>512	>512	512	512	>512
***P.aeruginosa*** l	>512	>512	>512	>512	>512	>512	>512	>512	>512	>512	>512
***P.aeruginosa*** m	512	>512	>512	>512	>512	>512	512	>512	>512	>512	>512
***S. maltophilia*** n	512	>512	>512	>512	>512	>512	>512	>512	>512	>512	>512
***A. baumannii*** o	512	>512	>512	>512	>512	>512	>512	>512	>512	>512	>512
***K.pneumoniae*** p	512	>512	>512	>512	>512	>512	512	>512	512	512	>512

a MIC, reported in µg/mL.

b ATCC 29213.

c ATCC 33592.

d 81388 CANWARD 2008.

e CAN-ICU 61589.

f ATCC 29212.

g ATCC 27270.

h ATCC 49619.

i ATCC 25922.

j CAN-ICU 61714.

k CAN-ICU 63074.

l ATCC 27853.

m CAN-ICU 62308.

n CAN-ICU 62584.

o CAN-ICU 63169.

p ATCC 13883.

**Table 5:** Antimicrobial testing of N_lys_N_lys_N_lys_ based lipopeptoids in the presence of 4% bovine serum albumin.

Compound Organism	CTAC	C11-N_lys_N_lys_N_lys_	C14-N_lys_N_lys_N_lys_	C16-N_lys_N_lys_N_lys_	C20-N_lys_N_lys_N_lys_	F11-N_lys_N_lys_N_lys_	C11-N_harg_N_harg_N_harg_	C14-N_harg_N_harg_N_harg_	C16-N_harg_N_harg_N_harg_	C20-N_harg_N_harg_N_harg_
***S.aureus*** b	32	>512	512	512	128	128	512	256	512	256
**MRSA** c	32	>512	512	>512	256	256	512	512	512	256
**MSSE** d	16	512	512	512	128	128	512	512	256	256
**MRSE** e	128	512	512	512	128	256	512	256	512	256
***E. faecalis*** f	32	>512	512	>512	256	512	>512	512	512	512
***E. faecium*** g	64	512	512	>512	256	256	512	256	512	128
***S.pneumoniae*** h	8	>512	>512	512	512	512	>512	256	>256	256
***E.coli*** i	512	>512	>512	>512	256	256	>512	512	512	512
***E.coli*** j	512	>512	>512	>512	256	256	>512	512	512	512
***E.coli*** k	512	>512	>512	>512	256	512	>512	512	>512	512
***P.aeruginosa*** l	>512	>512	>512	>512	512	512	>512	>512	>512	512
***P.aeruginosa*** m	512	>512	512	>512	512	512	>512	512	>512	>512
***S. maltophilia*** n	512	>512	>512	>512	512	>512	>512	>512	>512	>512
***A. baumannii*** o	512	>512	>512	>512	512	>512	>512	>512	>512	>512
***K.pneumoniae*** p	512	>512	>512	>512	256	>512	>512	512	>512	>512

a MIC, reported in µg/mL.

b ATCC 29213.

c ATCC 33592.

d 81388 CANWARD 2008.

e CAN-ICU 61589.

f ATCC 29212.

g ATCC 27270.

h ATCC 49619.

i ATCC 25922.

j CAN-ICU 61714.

k CAN-ICU 63074.

l ATCC 27853.

m CAN-ICU 62308.

n CAN-ICU 62584.

o CAN-ICU 63169.

p ATCC 13883.

The lipopeptoids under investigation were similarly inhibited, with those based on the N_lys_GN_lys_ and N_harg_GN_harg_ scaffolds demonstrating only weak activity (MIC 256 - >512 µg/mL) against Gram positive bacteria when BSA was added to the testing solutions. The N_lys_N_lys_N_lys_ and N_harg_N_harg_N_harg_ series fared somewhat better, and **C20-N_lys_N_lys_N_lys_** was able to inhibit some Gram positive strains at a high concentration (MIC 128 µg/mL, *S. epidermidis*).

### Hemolytic Testing

The positive control **CTAC** was highly hemolytic, lysing 77% of the ovine erythrocytes at only 100 µg/mL (**Tables 2** **and** **3**). Hemolytic activity of the peptoids was proportional to their antimicrobial activity, though the most hemolytic peptoids, **C20-N_harg_GN_harg_** and **C20-N_harg_N_harg_N_harg_**, were only slightly less toxic than **CTAC** (72% hemolysis at 100 µg/mL).

## Discussion

### Lipopeptoid Design

**Figure 1:**
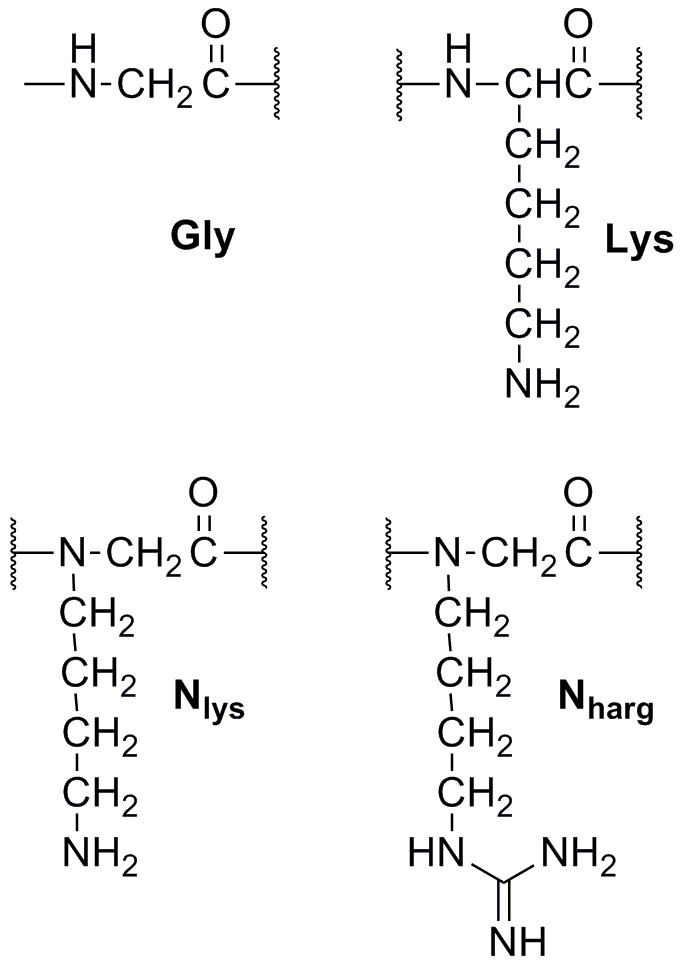
Peptoid residues with comparison amino acids. Lipid tails were attached at the N-terminus while all peptoids were amidated at their C-terminus.

To allow ready comparison with our previous work the peptoid sequences were modeled after KGK and KKK tripeptides [15], with the tails chosen for their previously demonstrated activity and selectivity (**Table 1**, **Figure 1**). Of note, one of our previously tested fluorinated tails was included, to test the effect of a hydrophobic and lipophobic moiety on peptoid toxicity and antimicrobial activity. Interested in the interplay between peptoid basicity and toxicity we also reacted nine of the ten initial lipopeptoids with a commercially available guanidylating reagent to create the homo-arginine peptoid analogues N_harg_GN_harg_ and N_harg_N_harg_N_harg_ (Figure 1). As the bacterial membrane is negatively charged, the stronger cationic character could potentially enhance antimicrobial activity.

### Antimicrobial Activity

With the cationic disinfectant **CTAC** as a positive control the antimicrobial activity of the lipopeptoids was assessed against a panel of clinically relevant bacteria. Several common reference laboratory bacterial strains were included as well, as quality control and for comparison to scaffolds from other research groups. **CTAC** was selected as it is a potent disinfectant [16], and unsurprisingly demonstrated good activity against the Gram positive bacteria (0.5–2 µg/mL) and moderate to limited activity against the Gram negative strains (16 to 128 µg/mL) in our panel.

While the most active of our lipopeptoids were unable to match the high activity of **CTAC**, several displayed comparable activity against Gram negative bacteria. In particular, both **C16-N_lys_GN_lys_** and **C16-N_harg_GN_harg_** inhibited all three strains of *E. coli* tested at 16 µg/mL (**Table 2**), despite their molecular mass being over twice that of **CTAC** (MIC 16–32 µg/mL).

The increased basicity of the N_harg_GN_harg_ and N_harg_N_harg_N_harg_ peptoid series appeared to convey a moderate increase in antimicrobial activity, though the effect varied depending on the lipid tail. When the N_lys_ analogue already demonstrated good activity, as in the case of **C16-N_lys_N_lys_N_lys_** (**Table 3**), the N_harg_ variant had little change in Gram positive activity but demonstrated improved activity against Gram negative strains (MIC decrease two-fold for all tested strains). The greatest improvement was observed in peptoids which were already weakly active, such as **C14-N_lys_GN_lys_** and **C14-N_lys_N_lys_N_lys_**. In these agents a two to four-fold improvement in MIC was observed against nearly every bacterial strain tested, though the activity always remained at or below that observed with **C16-N_lys_N_lys_N_lys_**.

When the N_lys_ variant of the peptoid was broadly inactive, increasing basicity resulted in similar improvements to the antimicrobial activity, but only against Gram positive bacteria. As both **C11-N_lys_GN_lys_** and **C11-N_lys_N_lys_N_lys_** were inactive against most of the Gram negative strains in our panels it is reasonable to assume that any improvement in activity remains beyond the limits of testing. Against expectations, increasing the basicity of even the most active peptoids did not increase their antimicrobial activity below 8 µg/mL. It is possible that there is a minimum concentration for peptoid activity or that peptoid activity is self-limiting due to aggregation or an unknown mechanism.

### Activity in the Presence of Bovine Serum Albumin

All CAMP analogues interact through non-specific interactions driven by their balance of hydrophobicity and charge, and the addition of hydrophobic proteins such as BSA is well known to cause a significant reduction in antimicrobial activity [10]. The positive control CTAC was no exception, with an approximately sixteen-fold reduction in activity against Gram positive bacteria when 4% BSA was added to the mixture. Activity against Gram negative bacteria, which are already naturally resilient to lysis because of their inner and outer cell membranes, was almost entirely eliminated.

Unfortunately, the peptoids were similarly limited. In the presence of BSA we observed nearly complete inhibition of both the N_lys_GN_lys_ and N_harg_GN_harg_ series, with the most active peptoid, F11-N_lys_GN_lys_, demonstrating only limited activity against several Gram positive bacteria (MIC 128–256 µg/mL) (**Table 4**). In a strange twist, the activity of **C20-N_harg_GN_harg_** against *S. pneumoniae* actually appeared to increase in the presence of BSA, from 256 µg/mL to 64 µg/mL. As this appears out of line with the results against other bacterial strains we are hesitant to draw significant conclusions in the absence of further testing.

Increasing the number of positive charges on the peptoids appeared to mitigate the inhibitory effect of BSA, with **C20-N_lys_N_lys_N_lys_**, **F11-N_lys_N_lys_N_lys_** and **C20-N_harg_N_harg_N_harg_** all demonstrating limited activity against *S. aureus* and *S. epidermidis* (MIC 128–256 µg/mL) (**Table 5**). Because the antimicrobial activity of these peptoids was similar to their N_lys_GN_lys_ and N_harg_GN_harg_ analogues it seems unlikely that the reduction in protein binding is a product of increased solubility, though it may stem from the particular conformation adopted by these peptoids. NMR analysis of all of the peptoids showed the presence of distinct rotameric states about the amide moieties, with restricted peptoid conformations in solution past 80°C (Supporting Information S1). The central glycine residue in the N_lys_GN_lys_ and N_harg_GN_harg_ peptoids allows them to freely rotate through their central core, and may aid in binding to the rigid BSA structure. In comparison, the N_lys_N_lys_N_lys_ and N_harg_N_harg_N_harg_ scaffolds are restricted throughout, and may prevent some of the conformations from effectively binding to BSA. Increasing the peptoid basicity by altering residues from N_lys_ to N_harg_ did not appear to reduce BSA binding, and may in fact have reduced selectivity for the bacterial membrane.

### Hemolytic Activity

Toxicity is a major concern with CAMPs, as their reliance on non-specific interactions often leads to disruption of zwitterionic mammalian membranes [8]. True to its strong antimicrobial activity, **CTAC** caused a high degree (77%) of lysis at 100 µg/mL, only slightly above its effective concentrations against Gram negative bacteria. The most toxic peptoids were also those with the strongest antimicrobial activity, though none were able to match the toxicity of CTAC. Seven of the eight peptoids with C16 or C20 tails lysed over 55% of the erythrocytes, with **C20-N_harg_GN_harg_** reaching 72% (**Tables 2** **and** **3**). Peptoids with homoarginine moieties were in general more toxic than their lysine analogue counterparts, despite potential repulsion with the zwitterionic eukaryotic membrane. This counter-intuitive increase is most visible with the peptoids **C14-N_lys_GN_lys_** and **C14-N_harg_GN_harg_** (2.9% vs 55.4% hemolysis at 100 µg/mL), and matches the corresponding increase in antimicrobial activity observed with these peptoids, as well as published work on longer lipopeptide sequences [12].

### Comparison to Previously Reported Lipopeptides

While the exact values may differ, antimicrobial activity between these lipopeptoids and their closest lipopeptide analogues follow similar trends, with both types of CAMPs having similar windows of activity and toxicity [15]. This reinforces the view that the primary activity of these CAMPs is determined by their physiochemical properties, not their specific structure, and suggests that previous research into lipopeptides can be directly applied to the development of new lipopeptoids.

However, the two scaffolds were not identical. Unlike the results obtained with the lipopeptide **C16-KGK** [15], no single lipopeptoid was significantly more effective than the others against Gram positive bacteria. Following from the previous conclusions about the balance of physical characteristics required for antimicrobial activity, this suggests that none of the peptoids in this study have the perfect balance of hydrophobicity and cationic charge required to inhibit the growth of Gram positive bacteria, with two or more of our compounds equally distant from the optimal lipopeptoid tail length. This is readily apparent with the peptoids **C16-N_harg_N_harg_N_harg_** and **C20-N_harg_N_harg_N_harg_**, which have nearly identical activity against each of the Gram positive bacteria in our survey.

Activity against Gram negative bacteria by contrast showed a preference for just a few peptoid sequences, with both **C16-N_lys_GN_lys_** and **C16-N_harg_GN_harg_** significantly more active than the peptoids with closely related tails. The balance of lipopeptoid hydrophobicity and charge optimal for activity against Gram positive bacteria appears to be different from that which is optimal against Gram negative bacteria, suggesting that there is a mild structural interaction with the exterior of the two types of bacteria.

Interestingly, both sets of compounds were significantly less active against the Gram positive bacteria *S. pneumoniae*, with activities more consistent with those displayed against Gram negative strains. In the context of the lipopeptides we previously attempted to rationalize this resistance as the result of an unexplored resistance mechanism, and can now eliminate the possibility that *S. pneumoniae* is expressing an endogenous protease, as the peptoid backbone is not susceptible proteolytic cleavage [13]. As both benzalknonium chloride and **CTAC** are able to maintain strong activity against *S. pneumoniae* a large scale alteration to the lipid bilayer also appears unlikely, suggesting that the poor lipopeptoid and lipopeptoid activity against *S. pneumoniae* results from localization of the agents away from the bacterial membrane perhaps via electrophilic, extracellular polymers such as teichuronic acid [9]. As benzalkonium chloride and **CTAC** contain quaternary amines they are extremely poor nucleophiles, unlikely to engage in hydrogen bonding.

### Conclusions

Nineteen new lipopeptoids have been prepared, with a variety of sequences and lipid tails. The antimicrobial activity of these compounds was assessed against a panel of clinically relevant and laboratory reference bacterial strains, including several drug resistant species. Compared to the cationic disinfectant **CTAC** the most active peptoids were less able to inhibit the growth of Gram positive bacteria, but were more active against Gram negative strains on a molar basis. Activity of all compounds in the presence of BSA was sharply reduced, though several peptoids retained limited activity against the Gram positive bacteria *S. aureus* and *S. epidermidis* (MIC 128–256 µg/mL).

Toxicity towards eukaryotic cells was found to correlate to antimicrobial activity, with the most active antimicrobials significantly hemolytic as well. This correlation was not observed in the weakly active peptoids however, with **C14-N_lys_GN_lys_** and **C14-N_lys_N_lys_N_lys_** able to inhibit Gram positive bacteria without significant hemolytic activity (MIC 16–64 µg/mL, <5% hemolysis at 100 µg/mL). Increasing the basicity of the compounds by replacing the lysine mimetic chains with homoarginine chains increased the activity of most of the peptoids tested, but in several cases resulted in a sharp increase in the hemolytic activity. Overall, the lipopeptoids produced were found to have antimicrobial activity similar to that of previously reported lipopeptides [15], with the potential to avoid proteolysis by both human serum proteins and endogenously expressed bacterial proteases.

## Materials and Methods

### Materials

Fmoc MBHA Rink Amide resin, Fmoc-Glycine-OH, TBTU and PyBop were purchased from Bachem (Switzerland). The fluorinated carboxylic acid was purchased from Fluorous Technologies Inc. (USA); Boc anhydride was purchased from AK Scintific Inc. (USA). Carboxylic acids with hydrocarbon tails and all other solvents and reagents were purchased from Sigma-Adrich (USA) at reagent grade and used without further purification.

### Peptide Synthesis

The lipopeptoid backbone was synthesized on solid phase, using standard chemical techniques [13], [17]. Aminated peptoids were purified using reverse-phase flash chromatography, with part of the yield then exposed to N,N′-diBoc-N′′-triflylguanidine to produce the guanidylated derivatives according to previously published techniques [18], [19]. Purity was confirmed with a mixture of ^1^H and ^13^C NMR on a Bruker AMX-500 spectrometer and ESI-MS on a Varian 500-MS IT Mass Spectrometer.

### Antimicrobial Activity

Antimicrobial activity of the purified lipopeptoids was determined without replication against a panel of clinically relevant and standard reference bacterial strains according to CLSI macrobroth standards [20]. Stock solutions at 512 µg/mL were prepared in water, with DMSO as needed, and testing was performed in glass test tubes using Muller-Hinton broth and bacteria adjusted to 5 × 10^5^ CFU/mL. Bacteria were incubated with the lipopeptoid of interest for 24 hr at 37°C prior to reading.

The bacteria Staphylococcus aureus ATCC 29213, methicillin-resistant S. aureus ATCC 33592, Staphylococcus epidermidis ATCC 14990, Enterococcus faecalis ATCC 29212, E. faecium ATCC 27270, Streptococcus pneumoniae ATCC 49619, Escherichia coli ATCC 25922, Pseudomonas aeruginosa ATCC 27853, and Klebsiella pneumoniae ATCC 13883 were acquired from the American Type Culture Collection (ATCC) and used as quality controls. The clinical strains methicillin-resistant S. epidermidis (MRSE) CAN-ICU 61589, E. coli CAN-ICU 61714, E. coli CAN-ICU 63074, P. aeruginosa CAN-ICU 62308, Stenotrophomonas maltophilia CAN-ICU 62584, Acinetobacter baumannii CAN-ICU 63169 were obtained from hospitals across Canada as part of the CAN-ICU studies [21], while methicillin-susceptible S. epidermidis (MSSE) 81388 was obtained from the 2008 CANWARD study [22].

### Haemolytic Activity

Mammalian cell toxicity was determined by measuring lysis of ovine erythrocytes, a standard model for human cell toxicity. Cells were prewashed with Tris buffered saline and then incubated with a variety of lipopeptoid concentrations for 30 minutes. Following centrifugation, lysis was evaluated by testing the absorbance of the solution at 540 nm, with 0.5% NH_4_OH used as a positive control [23].

## Supporting Information

Supporting Information S1 **Full biological methods, chemical synthesis and lipopeptoid spectra with Supporting** **Figure 1** **.**
(DOCX)
