# Supplementary material for: Guanidylation and Tail Effects in Cationic Antimicrobial Lipopeptoids
Source: PLoS One. 2012 Jul 23;7(7):e41141. doi: 10.1371/journal.pone.0041141 (PMC3402541; doi:10.1371/journal.pone.0041141)
Supplement: Supporting Information S1 — Full biological methods, chemical synthesis and lipopeptoid spectra with Supporting Figure 1 . (DOCX) [file pone.0041141.s001.docx]

**Supporting Information S1**

# Guanidylation and Tail Effects in Cationic Antimicrobial Lipopeptoids

Brandon Findlay, George G. Zhanel and Frank Schweizer*

*Departments of Chemistry and Medical Microbiology, University of Manitoba, Winnipeg,*

*Manitoba, R3T 2N2 Canada*

Contents

[Guanidylation and Tail Effects in Cationic Antimicrobial Lipopeptoids 1](#_Toc320533317)

[In vitro tests: 1](#_Toc320533318)

[Bacterial Isolates 2](#_Toc320533319)

[Antimicrobial susceptibilities 2](#_Toc320533320)

[Haemolytic Assays 2](#_Toc320533321)

[General Procedures 2](#_Toc320533322)

[t-Butyl (4-aminobutyl)carbamate Synthesis 3](#_Toc320533323)

[Fmoc Deprotection 3](#_Toc320533324)

[Peptoid residue synthesis 3](#_Toc320533325)

[Amino Acid Coupling 4](#_Toc320533326)

[Carboxylic Acid Coupling 4](#_Toc320533327)

[Cleavage from Rink Amide MBHA Resin 4](#_Toc320533328)

[Purification of Lipopeptoids 4](#_Toc320533329)

[Lipopeptoid Rotameric States 5](#_Toc320533330)

[Lipopeptoid Spectral Data 6](#_Toc320533331)

[Supplementary Materials References 13](#_Toc320533332)

## In vitro tests:

### Bacterial Isolates

Pathogenic bacteria were obtained during either the Canadian National Intensive Care Unit (CAN-ICU) study (1) or as part of the later CANWARD Canadian national surveillance study (2). The CAN-ICU study included 19 medical centres from across Canada with active ICUs. From September 2005 to June 2006, inclusive, each centre was asked to collect a maximium of 300 consecutive isolates obtained from clinical specimens such as blood, urine, wounds/tissues and respiratory samples (one pathogen per cultured site per patient) originating from their ICU patients. The 4180 isolates obtained corresponded to 2580 patients (1.62 isolates/patient). Study sites were requested to provide only “clinically significant” specimens, originating from patients with a presumed infectious disease. Isolates were delivered to the reference library (Health Sciences Centre, Winnipeg, Canada) on Amies charcoal swabs, then subcultured onto appropriate media and stocked in skim milk at -80°C until the minimum inhibitory concentration (MIC) testing was carried out.

### Antimicrobial susceptibilities

After subculturing the relevant bacteria twice from frozen stock, *in vitro* activities of the antimicrobials were determined by macrobroth dilution methodology in accordance with the Clinical and Laboratory Standards Institute (CLSI) guidelines (3). The MICs were determined using glass test tubes (2 mL/tube) containing doubling antimicrobial dilutions of cation adjusted Mueller-Hinton broth inoculated to achieve a final concentration of approximately 5 x 10^5^ CFU/mL, incubated in ambient air for 24 hr prior to reading. Colony counts were performed periodically to confirm inocula. The ATCC organisms *Staphylococcus pneumoniae* ATCC 49619, *Staphylococcus aureus* ATCC 29213, *Enterococcus faecalis* ATCC 29212, *Escherichia coli* ATCC 25922, and *Pseudomonas aeruginosa* ATCC 27853 were used as quality control and to allow easy comparison to previously tested antimicrobials. Each test in this series was performed without replication. In our experience with cationic amphiphiles the results are accurate to within a single doubling.

### Haemolytic Assays

Toxicity to mammalian cells was determined using a sheep red blood cell (erythrocyte) haemolytic assay (4). Erythrocytes were washed and resuspended in Tris buffered saline prior to use, at a concentration of 1.46 - 4.5 x 10^9^ cells/mL (on average 3.08 x 10^9^ cells/mL). The cell suspension was diluted with varying concentrations of antimicrobials, from 50 μg/mL to 1 mg/mL and incubated for thirty minutes (final erythrocyte concentration 4.08 x 10^8^ cells/mL). The samples were centrifuged following treatment and the absorbance of the supernatant was measured at 540 nm. A solution of 0.5% ammonium chloride was used as a positive control, with normal buffered solution as the negative control. The toxicity was assessed as a function of percent haemolysis.

## General Procedures

Reagents and solvents were purchased from commercially available sources and used without purification, unless otherwise noted. Fluorinated carboxylic acids were purchased from Fluorous Technologies Incorporated. Flash chromatography was performed using silica gel (Silicycle 23 -60 um) using standard techniques. ^1^H and ^13^C NMR were recorded on a Bruker AMX-500 or Bruker AMX-300 spectrometer in the noted solvents. Chemical shifts (δ) are reported in parts per million relative to tetramethylsilane. Compounds were visualized with either a mix of ninhydrin and acetic acid in ethanol, after spotting onto glass backed TLC plates. Low-resolution mass spectra (ESI+) were obtained on a Varian 500-MS IT Mass Spectrometer. All compounds were determined to be ≥90% pure by NMR prior to bacterial testing. Rink Amide MBHA resin was used to prepare each peptide and was swelled in DMF for a minimum of thirty minutes prior to initial deprotection.

### t-Butyl (4-aminobutyl)carbamate Synthesis

To a rapidly stirring solution of 1,4-diaminobutane (34.0 mL, 3 eq) and triethylamine (25.0 mL) in ice cold methanol (225 mL) was added a solution of Boc anhydride (24.9 g, 1 eq) in methanol (50 mL) over a period of 1 hr. The solution was allowed to gradually warm to room temperature and stir overnight. The methanol and triethylamine was then removed, and water added. Acetic acid was added (6.5 mL, 1.1 eq), and the solution extracted twice with diethyl ether. The aqueous layer was then basified with sodium carbonate and extracted twice with DCM. The DCM layers were combined, washed with 10% NaCO_3_, and concentrated. t-Butyl (4-aminobutyl)carbamate was obtained as a light yellow oil and used without further purification (10.4g, 49%).

### Fmoc Deprotection

To deblock the Rink amide resin prior to functionalization and to remove the Fmoc protecting group of the glycine residue following attachment to the growing peptoid chain, DMF:Piperidine (4:1) was added to pre-swelled resin, until it reached a level approximately three times the height of the bead bed, and the beads were gently agitated by a steady stream of air for forty minutes. The DMF:Piperidine mixture was then drained and the deprotecting process was repeated. The beads were then washed successively three times with DMF, then DCM, then DMF again, and a small sample of the beads was removed. This sample was treated with equal volumes of 2% chloranil and 2% acetaldehyde in DMF and successful deprotection was observed by the beads turning bright red.

### Peptoid residue synthesis

Following established procedure (5), beads containing unprotected amine moieties was added diisopropylcarbodiimide (DIC) (16.6 equivalents) and 2-bromoacetic acid (20 equivalents) in DMF. The mixture was agitated with a steady stream of nitrogen gas for thirty minutes, during which time a light yellow foam developed. The reactants were then drained off, and the resin was washed three times with DMF, three times with DCM and three times with NMP. A solution of tert-butyl (4-aminobutyl)carbamate (20 equivalents) in NMP was then added and agitated for ninety minutes. Once again the reaction mixture was drained and the beads were washed three times successively with NMP, DCM and DMF. At this point the chloranil test registered positive, with blue-green beads. Addition of bromoacetic acid could then be repeated for the NlysNlysNlys based residues, or Fmoc-Gly-OH added, as appropriate.

### Amino Acid Coupling

To the growing peptoid residues was added Fmoc protected glycine (3 equivalents), TBTU (3 equivalents) and Hunig’s Base (8 equivalents) in DMF. The mixtures were premixed at least three minutes prior to addition to ensure effective activation of the carboxylic acids. The solution was then gently agitated for at least three hours, after which time the solvent was drained and the beads were successively washed with 3x DMF, DCM and DMF. Completion of the reaction was verified through the chloranil test, as successfully reacted beads would not change colour in the presence of equal quantities of 2% chloranil and 2% acetaldehyde in DMF.

### Carboxylic Acid Coupling

As in the coupling of the glycine residues, hydrophobic tails were attached to the resin through the use of a mixture containing the carboxylic acid (3 equivalents), TBTU (3 equivalents) and Hunig’s Base (8 equivalents) in DMF. Because the activated fluorous carboxylic acids had extremely poor solubility in DMF they were instead premixed in DCM, with Pybop (3 equivalents) used as the activator. The quantity of Hunig’s base was unchanged (8 equivalents). Once the coupling mix was added the beads were gently agitated for approximately three hours, after which the chloranil test demonstrated that the coupling was complete.

### Cleavage from Rink Amide MBHA Resin

Resin containing the fully protected, complete peptoids was rinsed three times with DCM to remove any residual DMF, then dried under a steady vacuum. An acidic solution of TFA:Water:TIPS (95:2.5:2.5) was then added and the beads were agitated for a minimum of two hours. The TFA was filtered off and concentrated via evaporation under reduced pressure to yield crude lipopeptoids.

### Purification of Lipopeptoids

Peptoids were taken up in a minimum quantity of water and loaded onto a column containing C18 functionalized silica gel. The peptoids were then eluted by washing the column successively with distilled water (2.5 CV), 50% MeOH in water (2 CV), 75% MeOH in water (2 CV) and MeOH (3 CV). All solvents were stored in glass bottles, and acidified with 0.1% TFA. After three or more compounds had been purified with the column the column was washed with DCM and 1% TFA in MeOH. Fractions containing the peptoids of interest were collected and the solvent was removed via the steady passage of air at atmospheric pressure.

## Lipopeptoid Rotameric States

C14-NlysNlysNlys: ^1^H NMR (500 Mhz, D2O). Shifts were normalized by adjusting the HDO peak according to previously reported values (6). Glycine hydrogens are found from 4.0 – 4.5ppm and α-hydrogens of the lipid carbonyl are visible from 2.1 – 2.5ppm.


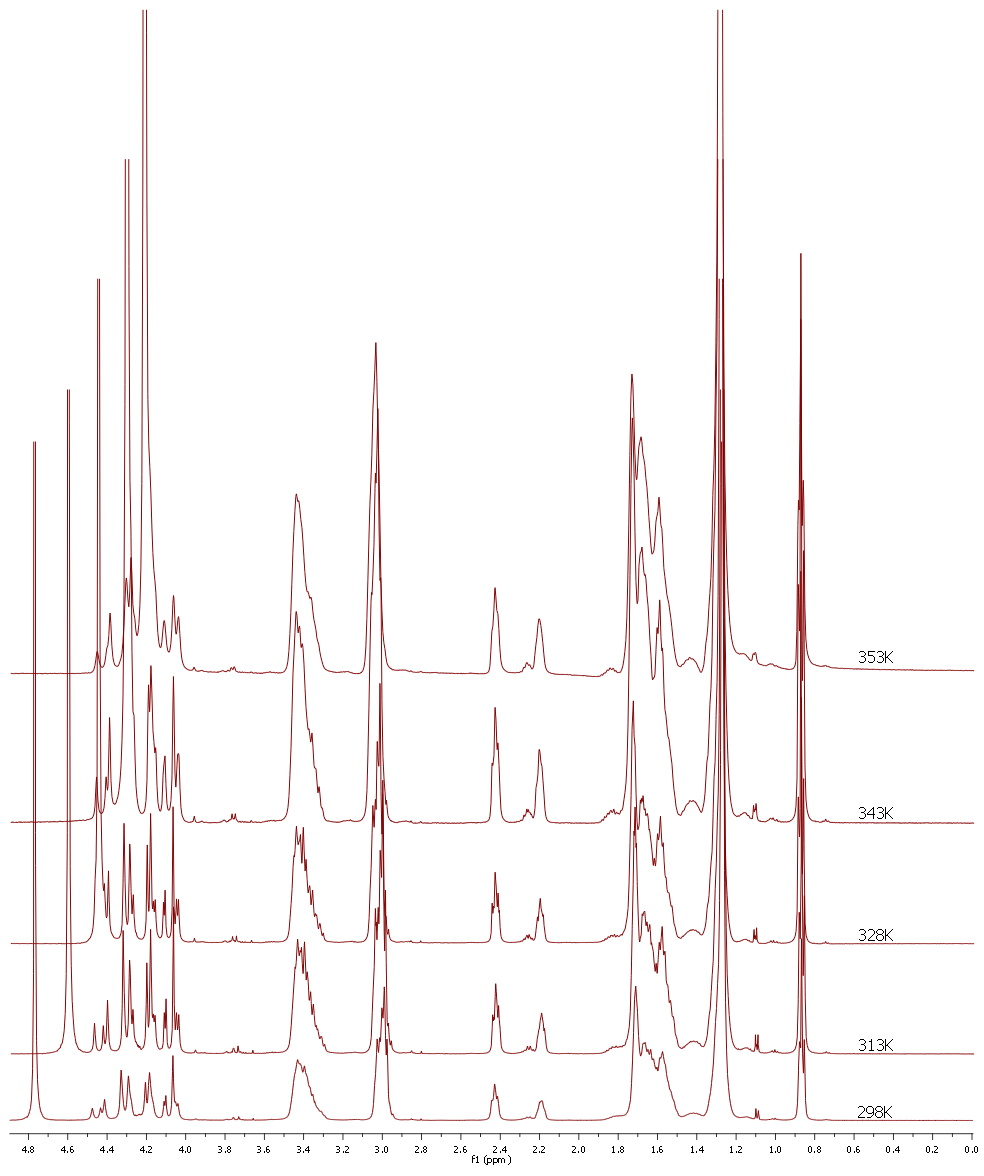


## Supporting Figure 1. Lipopeptoid rotameric states.

## Lipopeptoid Spectral Data

C11-NlysGNlys

^1^H NMR (500 MHz, MeOD, mixture of rotamers) δ 4.27 – 3.95 (m, 6H), 3.51 – 3.34 (m, 4H), 3.04 – 2.88 (m, 4H), 2.50 – 2.40 (m, 1H), 2.37 – 2.27 (m, 1H), 1.79 – 1.52 (m, 10H), 1.45 – 1.21 (m, 14H), 0.90 (t, J = 6.9, 3H). ^13^C NMR (126 MHz, MeOD, mixture of rotamers) δ 176.9, 176.4, 176.4, 173.8, 173.7, 173.0, 172.0, 171.8, 171.8, 171.8, 171.6, 171.6, 171.2, 171.1, 51.9, 50.7, 50.4, 50.2, 48.4, 48.3, 47.9, 47.9, 42.2, 42.2, 42.0, 40.6, 40.5, 34.3, 34.3, 33.9, 33.2, 30.8, 30.8, 30.8, 30.7, 30.6, 30.6, 30.6, 30.6, 26.7, 26.5, 26.4, 25.9, 25.9, 25.8, 25.5, 25.4, 23.9, 14.6. MS (ES) Calc. for C_25_H_51_N_6_O_4_ (M+H)^+^: 499.4. Found 499.6.

C14-NlysGNlys

^1^H NMR (500 MHz, MeOD, mixture of rotamers) δ 4.23 – 3.99 (m, 6H), 3.50 – 3.36 (m, 4H), 3.03 – 2.88 (m, 4H), 2.49 – 2.40 (m, 1H), 2.37 – 2.29 (m, 1H), 1.78 – 1.51 (m, 10H), 1.44 – 1.19 (m, 20H), 0.90 (t, J = 6.9, 3H). ^13^C NMR (126 MHz, MeOD, mixture of rotamers) δ 176.9, 176.4, 173.8, 173.7, 173.0, 172.0, 171.8, 171.8, 171.8, 171.6, 171.5, 171.2, 171.1, 51.9, 50.7, 50.4, 50.2, 48.4, 48.2, 47.9, 47.8, 42.2, 42.2, 42.0, 40.6, 40.5, 40.5, 34.3, 34.3, 33.9, 33.2, 30.9, 30.9, 30.8, 30.8, 30.6, 30.6, 26.7, 26.5, 26.5, 26.4, 25.9, 25.9, 25.8, 25.5, 25.4, 23.9, 21.6, 14.6. MS (ES) Calc. for C_28_H_57_N_6_O_4_ (M+H)^+^: 541.4. Found 541.7.

C16-NlysGNlys

^1^H NMR (500 MHz, MeOD, mixture of rotamers) δ 4.25 – 3.96 (m, 6H), 3.51 – 3.34 (m, 4H), 3.05 – 2.85 (m, 4H), 2.45 (t, J = 7.3, 1H), 2.33 (t, J = 7.3, 1H), 1.77 – 1.54 (m, 10H), 1.29 (s, 24H), 0.90 (t, J = 6.8, 3H). ^13^C NMR (126 MHz, MeOD, mixture of rotamers) δ 176.9, 176.4, 176.3, 173.8, 173.8, 173.0, 172.0, 171.8, 171.8, 171.8, 171.6, 171.6, 171.2, 171.1, 51.9, 50.7, 50.4, 50.2, 48.4, 48.2, 47.9, 47.9, 42.2, 42.2, 42.0, 40.6, 40.5, 40.5, 34.3, 34.3, 33.9, 33.2, 30.9, 30.9, 30.8, 30.8, 30.6, 30.6, 26.7, 26.5, 26.5, 26.4, 25.9, 25.9, 25.8, 25.5, 25.4, 23.9, 14.6. MS (ES) Calc. for C_30_H_61_N_6_O_4_ (M+H)^+^: 569.5. Found 569.7.

C20-NlysGNlys

^1^H NMR (500 MHz, MeOD, mixture of rotamers) δ 4.22 – 3.97 (m, 6H), 3.45 (dd, J = 13.5, 6.6, 4H), 3.02 – 2.87 (m, 4H), 2.47 – 2.43 (m, 1H), 2.35 – 2.24 (m, 1H), 1.77 – 1.52 (m, 10H), 1.42 – 1.21 (m, 32H), 0.90 (t, J = 6.9, 3H). ^13^C NMR (126 MHz, MeOD, mixture of rotamers) δ 177.0, 176.4, 174.0, 173.8, 173.8, 173.8, 173.0, 172.0, 54.4, 51.9, 50.7, 50.4, 50.2, 48.4, 48.3, 47.9, 47.9, 47.9, 42.2, 42.2, 42.0, 40.6, 40.5, 40.5, 34.3, 34.3, 33.9, 33.9, 33.2, 30.9, 30.9, 30.8, 30.8, 30.6, 30.6, 26.8, 26.5, 26.5, 25.9, 25.9, 25.5, 25.4, 23.9, 14.6. MS (ES) Calc. for C_34_H_69_N_6_O_4_ (M+H) ^+^: 625.5. Found 625.7.

F11-NlysGNlys

^1^H NMR (500 MHz, MeOD, mixture of rotamers) δ 4.28 – 3.95 (m, 6H), 3.55 – 3.35 (m, 4H), 3.07 – 2.90 (m, 4H), 2.87 – 2.77 (m, 1H), 2.77 – 2.67 (m, 1H), 2.65 – 2.45 (m, 2H), 1.80 – 1.56 (m, 8H).

^13^C NMR (126 MHz, MeOD, mixture of rotamers) δ 173.8, 173.8, 173.6, 173.0, 172.9, 172.9, 171.9, 171.8, 171.7, 171.6, 171.4, 171.3, 171.1, 51.8, 51.8, 50.7, 50.6, 50.5, 50.3, 50.2, 50.0, 50.0, 48.4, 48.3, 48.2, 42.3, 42.2, 42.1, 42.0, 40.6, 40.5, 33.2, 30.9, 27.8, 26.5, 26.4, 25.9, 25.9, 25.8, 25.5, 25.4, 25.3, 25.1. MS (ES) Calc. for C_25_H_34_F_17_N_6_O_4_ (M+H) ^+^: 805.2. Found 805.5.

C11-NhargGNharg NBoc

^1^H NMR (500 MHz, CDCl3, mixture of rotamers) δ 11.47 (d, J = 10.2, 2H), 8.42 – 8.24 (m, 2H), 7.23 – 5.54 (m, 3H), 4.22 – 3.91 (m, 6H), 3.39 (dd, J = 14.9, 8.4, 8H), 2.30 (dt, J = 58.1, 7.4, 2H), 1.90 (s, 0H), 1.74 – 1.38 (m, 46H), 1.24 (s, 14H), 0.86 (t, J = 6.8, 3H). ^13^C NMR (126 MHz, CDCl3, mixture of rotamers) δ 174.4, 171.0, 170.0, 169.8, 169.1, 163.7, 163.7, 156.5, 156.4, 153.5, 153.5, 83.4, 79.6, 50.6, 50.5, 49.5, 48.4, 41.2, 40.5, 40.3, 33.1, 32.1, 29.8, 29.7, 29.7, 29.6, 29.5, 28.5, 28.3, 26.6, 26.6, 26.3, 26.2, 25.9, 25.4, 22.9, 14.3.

C11-NhargGNharg

^1^H NMR (500 MHz, MeOD, mixture of rotamers) δ 4.24 – 3.98 (m, 6H), 3.53 – 3.37 (m, 4H), 3.26 – 3.11 (m, 4H), 2.46 (t, J = 7.5, 1H), 2.34 (t, J = 7.5, 1H), 1.79 – 1.50 (m, 10H), 1.40 – 1.23 (m, 14H), 0.90 (t, J = 6.8, 3H). ^13^C NMR (126 MHz, MeOD, mixture of rotamers) δ 177.0, 177.0, 176.4, 173.8, 173.1, 173.0, 172.0, 171.9, 171.8, 171.7, 171.7, 171.6, 171.2, 171.0, 158.8, 158.8, 51.8, 50.8, 50.7, 50.4, 50.3, 50.3, 48.4, 47.9, 42.3, 42.3, 42.2, 42.2, 42.0, 34.3, 33.9, 33.2, 30.9, 30.8, 30.8, 30.8, 30.6, 30.6, 28.3, 27.2, 27.2, 27.1, 26.9, 26.7, 26.6, 26.5, 26.5, 25.7, 25.7, 25.6, 24.4, 23.9, 14.6. MS (ES) Calc. for C_27_H_55_N_10_O_4_ (M+H)^+^: 583.4. Found 583.8.

C14-NhargGNharg NBoc

^1^H NMR (500 MHz, CDCl3, mixture of rotamers) δ 11.55 – 11.35 (m, 2H), 8.45 – 8.22 (m, 2H), 7.22 – 5.57 (m, 3H), 4.21 – 3.86 (m, 6H), 3.52 – 3.27 (m, 8H), 2.42 – 2.12 (m, 2H), 1.76 – 1.38 (m, 46H), 1.38 – 1.14 (m, 20H), 0.86 (t, J = 6.9, 3H). ^13^C NMR (126 MHz, CDCl3, mixture of rotamers) δ 174.3, 171.0, 169.8, 169.6, 169.1, 163.7, 163.6, 156.4, 156.4, 153.5, 153.5, 153.4, 83.4, 79.6, 50.5, 50.4, 49.4, 48.4, 47.1, 47.0, 47.0, 41.4, 41.2, 40.4, 40.3, 33.1, 32.1, 29.9, 29.8, 29.7, 29.7, 29.6, 29.5, 28.5, 28.3, 26.6, 26.6, 26.3, 25.9, 25.4, 22.9, 14.3.

C14-NhargGNharg

^1^H NMR (500 MHz, MeOD, mixture of rotamers) δ 4.27 – 3.97 (m, 6H), 3.51 – 3.38 (m, 4H), 3.24 – 3.11 (m, 4H), 2.45 (t, J = 7.4, 1H), 2.33 (t, J = 7.4, 1H), 1.75 – 1.49 (m, 10H), 1.29 (s, 20H), 0.90 (t, J = 6.7, 3H). ^13^C NMR (126 MHz, MeOD, mixture of rotamers) δ 177.0, 177.0, 176.4, 173.8, 173.1, 173.0, 172.0, 171.9, 171.8, 171.7, 171.7, 171.6, 171.2, 171.0, 158.8, 158.8, 51.8, 50.7, 50.7, 50.4, 50.4, 50.3, 50.3, 50.0, 48.6, 48.4, 47.9, 42.3, 42.3, 42.2, 42.2, 42.0, 34.3, 33.9, 33.2, 30.9, 30.9, 30.8, 30.8, 30.8, 30.6, 30.6, 27.2, 27.2, 27.2, 27.1, 26.9, 26.7, 26.6, 26.5, 26.5, 25.7, 25.7, 25.6, 23.9, 14.6. MS (ES) Calc. for C_30_H_61_N_10_O_4 ­_(M+H)^+^: 625.5. Found 625.9.

C16-NhargGNharg NBoc

^1^H NMR (500 MHz, MeOD, mixture of rotamers) δ 4.26 – 3.97 (m, 6H), 3.52 – 3.33 (m, 8H), 2.39 (dt, J = 57.7, 7.5, 2H), 1.76 – 1.40 (m, 46H), 1.40 – 1.20 (m, 24H), 0.90 (t, J = 6.9, 3H). ^13^C NMR (126 MHz, MeOD, mixture of rotamers) δ 176.4, 173.9, 171.9, 171.6, 171.3, 171.1, 171.0, 164.7, 157.8, 157.7, 154.3, 84.6, 84.5, 80.5, 80.4, 50.3, 50.3, 50.2, 50.1, 48.2, 42.2, 42.1, 41.7, 41.7, 41.4, 41.4, 34.3, 33.8, 33.2, 31.0, 30.9, 30.8, 30.7, 30.6, 30.6, 28.8, 28.4, 27.7, 27.7, 27.6, 27.5, 27.5, 27.0, 26.9, 26.7, 26.6, 26.5, 25.9, 25.8, 23.9, 14.6.

C16-NhargGNharg

^1^H NMR (300 MHz, MeOD, mixture of rotamers) δ 4.27 – 3.98 (m, 6H), 3.54 – 3.38 (m, 4H), 3.29 – 3.13 (m, 4H), 2.48 (t, J = 7.5, 1H), 2.36 (t, J = 7.4, 1H), 1.80 – 1.51 (m, 10H), 1.31 (s, 24H), 0.93 (t, J = 6.7, 3H). ^13^C NMR (126 MHz, MeOD, mixture of rotamers) δ 177.1, 177.0, 176.4, 173.8, 173.1, 173.0, 172.0, 171.9, 171.8, 171.7, 171.7, 171.6, 171.2, 171.0, 158.8, 158.8, 51.8, 51.8, 50.8, 50.7, 50.4, 50.4, 50.3, 50.3, 48.4, 47.9, 42.3, 42.3, 42.3, 42.2, 42.0, 34.3, 33.9, 33.2, 30.9, 30.9, 30.8, 30.8, 30.8, 30.6, 30.6, 27.2, 27.2, 27.1, 26.9, 26.7, 26.6, 26.5, 26.5, 25.7, 25.7, 25.6, 23.9, 14.6. MS (ES) Calc. for C_32_H_65_N_10_O_4 ­_(M+H)^+^: 653.5. Found 653.8.

C20-NhargGNharg NBoc

^1^H NMR (500 MHz, CDCl3, mixture of rotamers) δ 11.48 (d, J = 10.3, 2H), 8.58 – 8.15 (m, 2H), 7.22 – 5.51 (m, 3H), 4.28 – 3.85 (m, 6H), 3.57 – 3.17 (m, 8H), 2.46 – 2.26 (m, 2H), 1.72 – 1.40 (m, 46H), 1.40 – 1.07 (m, 32H), 0.87 (t, J = 6.9, 3H). ^13^C NMR (126 MHz, CDCl3, mixture of rotamers) δ 174.4, 174.3, 173.9, 171.0, 170.7, 170.0, 169.9, 169.7, 169.1, 168.7, 168.6, 163.7, 163.7, 156.5, 156.4, 153.5, 153.5, 153.5, 83.4, 79.6, 51.8, 50.6, 50.6, 50.4, 50.2, 49.5, 49.2, 48.5, 48.3, 47.1, 47.0, 41.5, 41.2, 40.8, 40.5, 40.4, 40.3, 40.2, 33.5, 33.2, 32.1, 31.4, 29.9, 29.9, 29.9, 29.8, 29.7, 29.7, 29.6, 29.6, 28.5, 28.3, 26.8, 26.6, 26.3, 26.2, 25.9, 25.5, 25.3, 25.2, 24.9, 22.9, 14.3. MS (ES) Calc. for C_56_H_104_N_10_NaO_12 ­_(M+Na)^+^: 1131.8. Found 1132.4.

C20-NhargGNharg

^1^H NMR (500 MHz, MeOD, mixture of rotamers) δ 4.26 – 3.95 (m, 6H), 3.53 – 3.38 (m, 4H), 3.26 – 3.11 (m, 4H), 2.45 (t, J = 7.5, 1H), 2.33 (t, J = 7.5, 1H), 1.76 – 1.48 (m, 10H), 1.48 – 1.11 (m, 32H), 0.90 (t, J = 6.8, 3H). ^13^C NMR (126 MHz, MeOD, mixture of rotamers) δ 177.1, 177.0, 176.4, 173.8, 173.1, 172.0, 171.9, 171.8, 171.7, 171.7, 171.6, 171.2, 171.0, 158.8, 158.8, 51.8, 51.8, 50.8, 50.8, 50.7, 50.4, 50.4, 50.3, 50.3, 50.3, 47.9, 42.3, 42.3, 42.3, 42.0, 34.3, 33.9, 33.2, 30.9, 30.9, 30.8, 30.8, 30.8, 30.6, 30.6, 30.6, 27.3, 27.2, 27.2, 27.2, 27.1, 26.9, 26.7, 26.6, 26.5, 26.5, 25.7, 25.7, 25.7, 23.9, 14.6. MS (ES) Calc. for C_36_H_73_N_10_O_4 ­_(M+H)^+^: 709.6. Found 710.0.

F11-NhargGNharg Nboc

^1^H NMR (500 MHz, CDCl3, mixture of rotamers) δ 11.65 – 11.34 (m, 2H), 8.37 (s, 2H), 7.18 – 5.50 (m, 3H), 4.24 – 3.90 (m, 6H), 3.54 – 3.29 (m, 8H), 2.75 – 2.64 (m, 2H), 2.64 – 2.43 (m, 2H), 1.79 – 1.35 (m, 44H). ^13^C NMR (126 MHz, CDCl3, mixture of rotamers) δ 171.0, 171.0, 170.9, 170.9, 170.7, 170.0, 169.2, 169.1, 168.8, 168.7, 168.5, 163.7, 163.7, 156.5, 156.5, 156.4, 153.6, 153.5, 153.5, 83.5, 79.7, 79.6, 51.4, 50.5, 50.4, 50.3, 50.2, 49.3, 49.0, 48.4, 48.3, 47.5, 47.3, 46.9, 41.5, 41.2, 40.7, 40.4, 40.2, 40.2, 29.9, 29.8, 28.5, 28.5, 28.5, 28.3, 28.2, 28.2, 28.1, 27.1, 26.9, 26.6, 26.6, 26.1, 26.0, 25.9, 25.1, 25.0, 24.8, 24.5, 24.2. MS (ES) Calc. for C_47_H_69_F_17_N_10_NaO_12_ (M+Na)^+^: 1311.5. Found 1312.3.

F11-NhargGNharg

^1^H NMR (500 MHz, MeOD, mixture of rotamers) δ 4.27 – 3.94 (m, 6H), 3.53 – 3.39 (m, 4H), 3.26 – 3.12 (m, 4H), 2.86 – 2.78 (m, 1H), 2.71 (d, J = 6.4, 1H), 2.55 (dd, J = 16.8, 9.9, 2H), 1.65 (dd, J = 33.6, 19.8, 8H). ^13^C NMR (126 MHz, MeOD, mixture of rotamers) δ 173.8, 173.8, 173.7, 173.7, 173.1, 173.0, 172.9, 171.9, 171.9, 171.7, 171.7, 171.5, 171.4, 171.2, 171.0, 158.8, 158.8, 51.7, 50.8, 50.7, 50.5, 50.5, 50.3, 50.3, 50.1, 50.0, 48.5, 48.4, 48.4, 42.3, 42.3, 42.2, 42.1, 42.0, 27.8, 27.7, 27.3, 27.2, 27.2, 27.1, 26.7, 26.7, 26.7, 25.7, 25.7, 25.3, 25.1. MS (ES) Calc. for C_77_H_39_F_17_N_10_O_12_ (M+2H)^2+^: 445.3. Found 445.5.

C11-NlysNlysNlys

^1^H NMR (500 MHz, MeOD, mixture of rotamers) δ 4.52 – 3.99 (m, 6H), 3.55 – 3.33 (m, 6H), 3.05 – 2.87 (m, 6H), 2.50 – 2.39 (m, 1H), 2.30 – 2.16 (m, 1H), 1.80 – 1.48 (m, 14H), 1.41 – 1.22 (m, 14H), 0.90 (t, J = 6.9, 3H).^13^C NMR (126 MHz, MeOD, mixture of rotamers) δ 177.5, 177.5, 177.2, 176.3, 173.0, 172.1, 172.1, 171.6, 171.4, 51.0, 50.3, 47.8, 40.5, 40.2, 34.3, 33.9, 33.2, 30.8, 30.8, 30.6, 27.0, 26.6, 26.6, 26.5, 26.4, 26.0, 25.9, 25.9, 25.8, 25.8, 25.7, 25.6, 25.4, 25.3, 23.9, 14.6. MS (ES) Calc. for C_29_H_60_N_7_O_4_ (M+H)^+^: 570.5. Found 570.6.

C14-NlysNlysNlys

^1^H NMR (500 MHz, MeOD, mixture of rotamers) δ 4.51 – 3.98 (m, 6H), 3.51 – 3.32 (m, 6H), 3.06 – 2.85 (m, 6H), 2.51 – 2.37 (m, 1H), 2.28 – 2.15 (m, 1H), 1.78 – 1.45 (m, 14H), 1.45 – 1.19 (m, 20H), 0.90 (t, J = 6.9, 3H). ^13^C NMR (126 MHz, MeOD, mixture of rotamers) δ 177.5, 176.2, 173.7, 173.0, 173.0, 172.1, 172.1, 171.6, 171.4, 171.1, 171.0, 170.6, 51.1, 50.3, 47.8, 40.6, 40.5, 40.5, 40.4, 34.3, 34.2, 33.9, 33.2, 30.9, 30.9, 30.8, 30.6, 26.6, 26.6, 26.5, 26.5, 26.5, 26.4, 26.0, 25.9, 25.9, 25.9, 25.8, 25.8, 25.6, 25.5, 23.9, 14.6. MS (ES) Calc. for C_32_H­_66_N_7_O_4_ (M+H)^+^: 612.5. Found 612.7.

C16-NlysNlysNlys

^1^H NMR (500 MHz, MeOD, mixture of rotamers) δ 4.51 – 3.97 (m, 6H), 3.50 – 3.33 (m, 6H), 3.07 – 2.89 (m, 6H), 2.49 – 2.37 (m, 1H), 2.29 – 2.14 (m, 1H), 1.81 – 1.49 (m, 14H), 1.28 (s, 24H), 0.90 (t, J = 6.9, 3H). ^13^C NMR (126 MHz, MeOD, mixture of rotamers) δ 177.5, 177.5, 177.2, 176.2, 173.4, 173.3, 173.0, 172.1, 172.1, 171.6, 171.6, 171.4, 170.9, 170.7, 170.6, 51.0, 50.4, 50.3, 50.2, 47.8, 40.6, 40.6, 40.5, 40.5, 40.4, 34.3, 34.2, 33.9, 33.8, 33.2, 30.9, 30.9, 30.8, 30.6, 29.1, 26.6, 26.6, 26.5, 26.4, 26.4, 26.0, 25.9, 25.9, 25.8, 25.8, 25.6, 25.6, 25.4, 25.4, 25.3, 23.9, 14.6. MS (ES) Calc. for C_34_H_70_N_7_O_4_ (M+H)^+^: 640.5. Found 640.8.

C20-NlysNlysNlys

^1^H NMR (500 MHz, MeOD, mixture of rotamers) δ 4.53 – 3.97 (m, 6H), 3.51 – 3.33 (m, 6H), 3.06 – 2.87 (m, 6H), 2.48 – 2.39 (m, 1H), 2.27 – 2.17 (m, 1H), 1.80 – 1.47 (m, 14H), 1.31 (s, J = 25.5, 32H), 0.90 (t, J = 7.0, 3H). ^13^C NMR (126 MHz, MeOD, mixture of rotamers) δ 177.5, 177.4, 177.2, 176.2, 176.2, 176.2, 176.1, 172.1, 172.1, 171.6, 171.4, 51.0, 50.8, 50.8, 50.7, 50.3, 47.8, 40.6, 40.6, 40.5, 40.5, 40.4, 34.3, 34.2, 34.2, 33.9, 33.9, 33.8, 33.2, 30.9, 30.9, 30.8, 30.8, 30.6, 26.6, 26.6, 26.6, 26.5, 26.5, 26.4, 26.0, 25.9, 25.9, 25.9, 25.8, 25.8, 25.6, 25.4, 23.9, 14.6. MS (ES) Calc. for C_38_H_78_N_7_O_4_ (M+H)^+^: 696.6. Found 696.9.

F11-NlysNlysNlys

^1^H NMR (500 MHz, MeOD, mixture of rotamers) δ 4.55 – 3.93 (m, 6H), 3.54 – 3.34 (m, 6H), 3.06 – 2.89 (m, 6H), 2.86 – 2.76 (m, 1H), 2.70 – 2.37 (m, 3H), 1.85 – 1.51 (m, 12H). MS (ES) Calc. for C_29_H_43_F_17_N_7_O_4_ (M+H)^+^: 876.3. Found 876.6.

C11-NhargNhargNharg NBoc

^1^H NMR (500 MHz, CDCl3, mixture of rotamers) δ 11.49 (s, 3H), 8.35 (s, 3H), 4.53 – 3.86 (m, 6H), 3.44 (s, 12H), 2.46 – 2.18 (m, 2H), 1.80 – 1.01 (m, 82H), 0.95 – 0.78 (m, 3H). ^13^C NMR (126 MHz, CDCl3, mixture of rotamers) δ 163.8, 156.4, 156.4, 153.6, 153.5, 148.9, 83.6, 83.4, 83.4, 79.6, 79.6, 79.5, 50.9, 49.5, 49.3, 48.8, 47.9, 47.4, 46.9, 40.5, 40.5, 40.3, 40.2, 33.0, 32.1, 31.5, 29.8, 29.7, 29.5, 28.5, 28.3, 28.2, 26.8, 26.7, 26.6, 26.2, 26.1, 25.4, 24.9, 22.9, 14.3.

C11-NhargNhargNharg

^1^H NMR (500 MHz, MeOD, mixture of rotamers) δ 4.52 – 3.96 (m, 6H), 3.51 – 3.33 (m, 6H), 3.27 – 3.08 (m, 6H), 2.43 (t, J = 7.4, 1H), 2.28 – 2.17 (m, 1H), 1.78 – 1.45 (m, 14H), 1.45 – 1.10 (m, 14H), 0.90 (t, J = 6.8, 3H). ^13^C NMR (126 MHz, MeOD, mixture of rotamers) δ 177.6, 176.2, 173.8, 173.1, 172.1, 171.7, 171.6, 171.4, 171.1, 170.7, 170.6, 158.9, 158.8, 158.8, 51.0, 50.7, 50.5, 50.4, 47.9, 47.9, 42.4, 42.3, 42.1, 34.3, 34.3, 33.9, 33.2, 33.2, 27.2, 26.8, 26.7, 26.6, 25.9, 25.7, 25.7, 25.6, 23.9, 14.6. MS (ES) Calc. for C_32_H_63_N_13_O_4 ­_(M+H)^+^: 696.5. Found 696.9.

C14-NhargNhargNharg NBoc

^1^H NMR (500 MHz, CDCl3, mixture of rotamers) δ 11.47 (s, 2H), 8.84 (s, 1H), 8.33 (d, J = 4.0, 3H), 4.43 – 3.86 (m, 6H), 3.50 – 3.25 (m, 12H), 2.31 (d, J = 7.7, 2H), 1.78 – 1.31 (m, 68H), 1.31 – 1.10 (m, 20H), 0.85 (t, J = 6.9, 3H). ^13^C NMR (126 MHz, CDCl3, mixture of rotamers) δ 173.7, 171.6, 169.4, 163.7, 163.7, 156.4, 153.5, 153.5, 153.4, 83.5, 83.3, 83.3, 79.5, 79.5, 50.8, 48.7, 40.5, 32.9, 32.1, 29.8, 29.8, 29.7, 29.7, 29.6, 29.5, 28.4, 28.2, 28.1, 28.0, 26.6, 25.3, 22.8, 14.3.

C14-NhargNhargNharg

^1^H NMR (500 MHz, MeOD, mixture of isomers) δ 4.52 – 4.00 (m, 6H), 3.51 – 3.33 (m, 6H), 3.26 – 3.11 (m, 6H), 2.52 – 2.35 (m, 1H), 2.30 – 2.14 (m, 1H), 1.88 – 1.47 (m, 14H), 1.29 (s, 20H), 0.90 (t, J = 6.8, 3H). ^13^C NMR (126 MHz, MeOD, mixture of rotamers) δ 177.6, 177.6, 177.3, 177.3, 176.9, 176.3, 176.2, 176.2, 173.9, 173.8, 173.4, 173.4, 173.1, 172.2, 172.2, 172.1, 172.1, 171.7, 171.6, 171.5, 171.1, 171.1, 171.0, 170.9, 170.9, 170.8, 170.7, 170.6, 158.9, 158.8, 51.0, 50.8, 50.7, 50.5, 50.4, 50.3, 50.1, 48.5, 48.1, 47.9, 42.4, 42.3, 42.3, 42.3, 41.9, 34.3, 34.3, 33.9, 33.2, 30.9, 30.8, 30.6, 29.4, 27.4, 27.3, 27.2, 27.2, 27.1, 27.1, 27.0, 26.9, 26.8, 26.8, 26.7, 26.6, 26.5, 26.4, 25.9, 25.9, 25.8, 25.7, 25.7, 25.7, 25.6, 23.9, 14.6. MS (ES) Calc. for C_35_H_72_N_13_O_4 ­_(M+H)^+^: 738.6. Found 739.0.

C16-NhargNhargNharg Nboc

^1^H NMR (500 MHz, CDCl3, mixture of isomers) δ 11.48 (s, 3H), 8.34 (s, 3H), 4.08 (d, J = 71.3, 6H), 3.41 (d, J = 22.4, 12H), 2.32 (s, 2H), 1.53 (d, J = 50.1, 92H), 0.87 (s, 3H). ^13^C NMR (126 MHz, CDCl3, mixture of isomers) δ 163.7, 156.5, 156.4, 156.4, 156.3, 153.5, 153.5, 153.4, 83.4, 83.3, 79.6, 79.5, 50.8, 49.5, 49.2, 48.8, 40.5, 33.0, 32.1, 29.9, 29.7, 29.7, 29.6, 28.5, 28.3, 28.2, 28.1, 26.7, 26.6, 26.1, 25.4, 22.9, 14.3.

C16-NhargNhargNharg

^1^H NMR (500 MHz, MeOD, mixture of rotamers) δ 4.50 – 3.96 (m, 6H), 3.51 – 3.34 (m, 6H), 3.25 – 3.13 (m, 6H), 2.50 – 2.36 (m, 1H), 2.30 – 2.18 (m, 1H), 1.82 – 1.46 (m, 14H), 1.46 – 1.21 (m, 24H), 0.90 (t, J = 6.9, 3H). ^13^C NMR (126 MHz, MeOD, mixture of rotamers) δ 177.6, 177.6, 176.3, 176.2, 176.2, 173.4, 173.4, 173.1, 172.2, 172.1, 172.1, 171.6, 171.5, 171.1, 171.1, 171.0, 170.9, 170.9, 170.8, 170.7, 170.6, 158.8, 158.8, 67.0, 51.0, 50.7, 50.5, 50.4, 50.3, 48.1, 47.9, 42.4, 42.4, 42.3, 42.3, 34.3, 34.3, 33.9, 33.2, 31.0, 30.9, 30.8, 30.8, 30.6, 27.4, 27.3, 27.2, 27.1, 26.9, 26.8, 26.7, 26.7, 26.6, 26.5, 26.4, 25.9, 25.7, 25.7, 25.6, 23.9, 14.6. MS (ES) Calc. for C_37_H_76_N_13_O_4 ­_(M+H)^+^: 766.6. Found 767.0.

C20-NhargNhargNharg Nboc

1H NMR (500 MHz, CDCl3, mixture of rotamers) δ 11.47 (s, 3H), 8.53 – 8.05 (m, 3H), 4.31 – 3.86 (m, 6H), 3.54 – 3.24 (m, 12H), 2.26 (dd, J = 62.9, 55.2, 2H), 1.85 – 1.04 (m, 100H), 0.86 (t, J = 6.8, 3H). ^13^C NMR (126 MHz, CDCl3, mixture of rotamers) δ 173.5, 169.4, 169.2, 163.5, 156.2, 153.3, 153.3, 83.2, 83.1, 79.4, 79.3, 51.1, 50.7, 49.3, 49.1, 49.1, 48.6, 48.6, 47.7, 47.2, 46.8, 40.4, 40.4, 40.3, 40.3, 40.3, 40.1, 40.1, 40.0, 32.8, 32.8, 31.9, 31.9, 29.8, 29.8, 29.7, 29.7, 29.7, 29.7, 29.6, 29.6, 29.6, 29.6, 29.5, 29.5, 29.4, 29.4, 28.3, 28.3, 28.1, 28.1, 28.0, 28.0, 26.6, 26.5, 26.5, 26.4, 25.9, 25.9, 25.2, 25.2, 22.7, 22.7, 14.1, 14.1.

C20-NhargNhargNharg

^1^H NMR (500 MHz, MeOD, mixture of rotamers) δ 4.51 – 4.00 (m, 6H), 3.48 – 3.34 (m, 6H), 3.27 – 3.15 (m, 6H), 2.48 – 2.37 (m, 1H), 2.27 – 2.18 (m, 1H), 1.80 – 1.46 (m, 14H), 1.42 – 1.21 (m, 32H), 0.90 (t, J = 6.9, 3H). ^13^C NMR (126 MHz, MeOD, mixture of rotamers) δ 177.6, 176.3, 176.2, 176.2, 173.4, 173.4, 173.1, 172.1, 171.6, 171.5, 171.1, 171.1, 158.8, 158.8, 67.1, 51.0, 50.5, 50.3, 47.9, 42.4, 42.3, 42.3, 34.3, 34.3, 33.9, 33.2, 30.9, 30.9, 30.8, 30.8, 30.6, 30.6, 27.4, 27.3, 27.2, 26.8, 26.7, 26.6, 26.4, 25.9, 25.8, 25.7, 25.6, 23.9, 14.6. MS (ES) Calc. for C_41_H_85_N_13_O_4 ­_(M+H)^2+^: 411.9. Found 412.1.

## Supplementary Materials References

1. Zhanel GG, DeCorby M, Laing N, Weshnoweski B, Vashisht R, Tailor F, et al. Antimicrobial-resistant pathogens in intensive care units in canada: Results of the canadian national intensive care unit (CAN-ICU) study, 2005-2006. Antimicrob Agents Chemother. 2008 Apr;52(4):1430-7.

2. Zhanel GG, Adam HJ, Low DE, Blondeau J, Decorby M, Karlowsky JA, et al. Antimicrobial susceptibility of 15,644 pathogens from canadian hospitals: Results of the CANWARD 2007-2009 study. Diagn Microbiol Infect Dis. 2011 Mar;69(3):291-306.

3. Rennie RP, Callihan DR, Barry AL, Krisher K, Munro SD, Sei K, et al. Protocols for evaluating dehydrated mueller-hinton agar; approved standard-second edition. In: CLSI; 2006. p. 1-29.

4. Dathe M, Schumann M, Wieprecht T, Winkler A, Beyermann M, Krause E, et al. Peptide helicity and membrane surface charge modulate the balance of electrostatic and hydrophobic interactions with lipid bilayers and biological membranes. Biochemistry. 1996 Sep 24;35(38):12612-22.

5. Chongsiriwatana NP, Patch JA, Czyzewski AM, Dohm MT, Ivankin A, Gidalevitz D, et al. Peptoids that mimic the structure, function, and mechanism of helical antimicrobial peptides. Proc Natl Acad Sci U S A. 2008 Feb 26;105(8):2794-9.

6. Gottlieb HE, Kotlyar V, Nudelman A. NMR chemical shifts of common laboratory solvents as trace impurities. J Org Chem. 1997 Oct 17;62(21):7512-5.
